# Deletion of mouse FXR gene disturbs multiple neurotransmitter systems and alters neurobehavior

**DOI:** 10.3389/fnbeh.2015.00070

**Published:** 2015-03-30

**Authors:** Fei Huang, Tingting Wang, Yunyi Lan, Li Yang, Weihong Pan, Yonghui Zhu, Boyang Lv, Yuting Wei, Hailian Shi, Hui Wu, Beibei Zhang, Jie Wang, Xiaofeng Duan, Zhibi Hu, Xiaojun Wu

**Affiliations:** ^1^The Ministry of Education Key Laboratory for Standardization of Chinese Medicines, the State Administration of TCM Key Laboratory for New Resources and Quality Evaluation of Chinese Medicine, Shanghai Key Laboratory of Complex Prescriptions, Institute of Chinese Materia Medica, Shanghai University of Traditional Chinese MedicineShanghai, China; ^2^Blood-Brain Barrier Group, Pennington Biomedical Research CenterBaton Rouge, LA, USA; ^3^Pharmacy Department, Shanghai East HospitalShanghai, China

**Keywords:** FXR, bile acid, neurobehavior, neurotransmitter, neurotransmission, emotion, memory, motor performance

## Abstract

Farnesoid X receptor (FXR) is a nuclear hormone receptor involved in bile acid synthesis and homeostasis. Dysfunction of FXR is involved in cholestasis and atherosclerosis. FXR is prevalent in liver, gallbladder, and intestine, but it is not yet clear whether it modulates neurobehavior. In the current study, we tested the hypothesis that mouse FXR deficiency affects a specific subset of neurotransmitters and results in an unique behavioral phenotype. The FXR knockout mice showed less depressive-like and anxiety-related behavior, but increased motor activity. They had impaired memory and reduced motor coordination. There were changes of glutamatergic, GABAergic, serotoninergic, and norepinephrinergic neurotransmission in either hippocampus or cerebellum. FXR deletion decreased the amount of the GABA synthesis enzyme GAD65 in hippocampus but increased GABA transporter GAT1 in cerebral cortex. FXR deletion increased serum concentrations of many bile acids, including taurodehydrocholic acid, taurocholic acid, deoxycholic acid (DCA), glycocholic acid (GCA), tauro-α-muricholic acid, tauro-ω-muricholic acid, and hyodeoxycholic acid (HDCA). There were also changes in brain concentrations of taurocholic acid, taurodehydrocholic acid, tauro-ω-muricholic acid, tauro-β-muricholic acid, deoxycholic acid, and lithocholic acid (LCA). Taken together, the results from studies with FXR knockout mice suggest that FXR contributes to the homeostasis of multiple neurotransmitter systems in different brain regions and modulates neurobehavior. The effect appears to be at least partially mediated by bile acids that are known to cross the blood-brain barrier (BBB) inducing potential neurotoxicity.

## Introduction

Farnesoid X receptor (FXR) is a member of the family of nuclear hormone receptors. Upon activation and nuclear translocation, FXR forms a heterodimer with retinoid X receptor (RXR) that binds to its cognate DNA response elements. FXR is a bile acid receptor (BAR) and plays a critical role in the maintenance of bile acid (BA) synthesis and homeostasis (Maruyama et al., 2002; Pircher et al., 2003). Activation of FXR inhibits hepatic acid biosynthesis and accelerates the transport of BAs from the intestinal lumen to the liver. On the contrary, *FXR* deletion results in disrupted bile acid homeostasis, shown by elevated serum bile acid, cholesterol, and triglycerides, as well as increased hepatic cholesterol and triglycerides (Sinal et al., 2000). FXR dysfunction contributes to many diseases, such as hepatic tumorigenesis (Kim et al., 2007b; Yang et al., 2007), intestinal diseases (Kim et al., 2007a), cholestasis (Stedman et al., 2006), atherosclerosis (Guo et al., 2006), and impaired liver regeneration (Huang et al., 2006; Fan et al., 2015).

Not surprisingly, FXR mRNA is most abundant in liver, ileum, and kidney, tissues that are exposed to high concentrations of BAs (Forman et al., 1995; Seol et al., 1995). This is consistent with its active involvement in BA synthesis, secretion, transport, absorption, conjugation, and detoxification. Unlike other nuclear hormone receptors including liver X receptor, retinoid X receptor and peroxisome proliferator activating receptor, *FXR* mRNA is not detectable in brain cells or capillary endothelial cells (Akanuma et al., 2008). In agreement with the reports, our unpublished data (Supplementary Figure 1) showed that *FXR* mRNA was not present in mouse brain. Moreover, one of the natural FXR ligands, chenodeoxycholic acid, does not appear to cross the normal mouse blood-brain barrier (BBB) (Jia et al., 2014). Therefore, FXR appears to be absent in mouse central nervous system (CNS). Perhaps for such a reason, whether FXR deletion alters mouse neurobehavior has not been reported yet.

Many hepatic diseases are associated with neurological presentations, such as anxiety (Tkachenko et al., 2013), depression (Suh et al., 2013), mania (Machado et al., 2008), and cognitive impairment (Monfort et al., 2007). Cholestasis is one of such disorders that can produce encephalopathy by deposition of neurotoxins in the brain (Garcia-Moreno et al., 2002). This in turn changes glutamatergic, GABAergic, and serotoninergic transmission and neuronal circuits (Cauli et al., 2009; Magen et al., 2010). Moreover, cholestasis can affect cytoarchitecture of the brain, including the hippocampus and inferiotemporal cortex.

FXR KO mice have been reported to show less obstructive cholestasis but increased serum BAs in comparison with normal mice (Stedman et al., 2006). Circulating serum BAs, such as DCA and CDCA, can increase permeability of the BBB via disruption of tight junctions (Quinn et al., 2014). Therefore, we speculated that FXR deletion may disrupt the BBB so as to increase the access of BAs and other toxins from blood to brain, which interferes with neurotransmission and leads to specific behavioral changes. To test this hypothesis, in the current study we examined the neurobehavior of FXR KO mice with their controls through a battery of behavioral tests, including open-field, elevated plus maze, forced-swimming, tail-suspension, passive avoidance, and rotarod. Neurotransmitters in the hippocampus, cerebellum, and prefrontal cortex of the mice were analyzed by LC-MS/MS. We also investigated the expression of relevant proteins involved in the synthesis, transport, and signaling of neurotransmitters by western blotting assay as well as serum and brain BAs by an UPLC-MS approach. The results indicate that FXR deletion changes many aspects of neurobehavior in mice linked to specific neurochemical changes. The novel findings extend our understanding of the role of FXR in modulation of mouse neurobehavior.

## Materials and methods

### Animals

FXR knockout (KO) mice were provided by the Laboratory of Metabolism in the Division of Basic Sciences of National Institutes of Health (Bethesda, MD, USA) and bred in the Experimental Animal Center of Shanghai University of Traditional Chinese Medicine (SHUTCM, Shanghai, China). The KO mice had normal weight gain, normal developmental milestones, and were fertile (Sinal et al., 2000). Since FXR null mice have been maintained on a C57BL/6 background, age matched wild-type C57BL/6 mice were used as the experimental controls. The behavioral tests were conducted when the mice were 4–5 months old. Each group contained 12 male mice that were group-housed with a 12 h light/12 h dark cycle at room temperature (25 ± 1°C) and fed regular rodent chow and water *ad libitum*. All experiments on animals were performed according to a protocol approved by the Institutional Animal Care and Use Committee of SHUTCM.

### Behavioral studies

**Figure 1:**
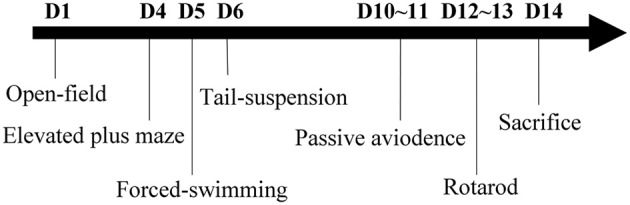
**Experimental paradigm of the behavioral tests**.

All behavioral tests were conducted during the light phase between 09:00 a.m. and 17:00 p.m. To avoid experimental bias, all behavioral observers were blind to the genotype of the test mice. The experimental paradigm for behavioral tests is shown as Figure 1. This includes open-field, elevated plus maze, forced-swimming, tail-suspension, passive avoidance, and rotarod. All tests were performed sequentially in 2 weeks with proper resting time to avoid fatigue. Moreover, the mice from different groups were tested in a random order. To avoid reciprocal influence among mice due to odor from feces and urine, the apparatus surfaces were cleaned with 10% ethanol among batches of individual subjects.

#### Open-field test (OFT)

Mice were placed in the center of a square field (50 × 50 cm) that was enclosed by white walls (height 40 cm) and well-illuminated (150 lux). Locomotor activity of individual mice was video-recorded for 5 min. The central and total distance traveled as well as the time spent in the central and peripheral areas were analyzed by a video-tracking system (Mobiledatum Inc., Shanghai, China).

#### Elevated plus maze test (EPMT)

The experiments were conducted in a dark room illuminated with a dim lamp (12-W fluorescent). The apparatus was elevated 60 cm from the floor, composed of a central platform (5 × 5 cm) connected with two open arms and two enclosed arms (30 × 5 cm). The mouse was placed in the central platform facing the open arms. Total time spent completely in the open arms and the number of exploring the open arms were recorded for 5 min.

#### Forced-swimming test (FST)

Mice were individually placed in a glass cylinder (tall 30 cm, diameter 20 cm) filled with water (height 20 cm) at ambient temperature. The animals were allowed to swim freely for 6 min, and the duration of immobility was video-recorded. Immobility was defined as time floating passively without any active movement in the last 4 min.

#### Tail-suspension test (TST)

Tails of mice were taped to a horizontal bar elevated 60 cm from the floor. The behavior of mice for 6 min and the duration of immobility in the last 4 min were video-recorded for final comparison. Immobility was defined as no active movements except for respiration.

#### Passive avoidance test (PAT)

The shuttle box used for PAT was divided into bright and dark chambers (30 × 30 × 30 cm for each one) by an opaque Plexiglas door. The bright chamber was illuminated by fluorescent light (approximately 500 lux). The floor of the box was made of stainless steel grids connected to an electric shock generator. At the beginning of training, the mice were placed in the bright chamber with their back toward the entrance of the dark one. Once mice entered the dark chamber, they immediately received an electric shock of 0.4 mA until they returned to the bright chamber. Each mouse was trained until it met a learning criterion of 300-s totally staying in the bright chamber. On the second day, the time for the passive avoidance response of the mice was evaluated in 300-s. The latency to enter the dark chamber as well as the number of entries was analyzed by a video-tracking system (Mobiledatum). The number of trials to criterion was used to measure PAT acquisition, and time before entering the dark chamber 24 h after training was used to measure PAT memory.

#### Rotarod test (RRT)

The rotarod test was performed using a Rota-Rod Treadmill (Mobiledatum). The mice were trained to learn to walk steadily on a horizontally oriented rod rotating at the speed of 15 rpm. On the test day, they were obliged to walk on the rod rotating at 35 rpm for a maximal 600 s. The duration for a mouse to stay on the rod was recorded. The trials were repeated for three times at 40 min intervals in 1 day. Finally, the data were averaged for statistical analysis.

### Neurotransmitter and bile acid analysis

Upon completion of all behavioral tests, the mice were anesthetized with 20% urethane and sacrificed. The hippocampus, cerebellum, and prefrontal cortex were dissected immediately on ice, snap frozen in liquid nitrogen and stored at −80°C until analysis. The concentrations of neurotransmitters including dopamine (DA), 5-hydroxy tryptamine (5-HT), γ-aminobutyric acid (GABA), glutamic acid (Glu), norepinephrine (NE), epinephrine (Epi), and 5-hydroxyindole acetic acid (5-HIAA) were determined by LC-MS/MS method described previously (Huang et al., 2014).

Blood samples were obtained from the retinal venous plexus of each mouse and stored at 4°C overnight. Whole serum was collected after centrifugation of whole blood at 845 g for 10 min at 4°C. Brain samples were prepared by homogenization of the whole brains in 1 ml of 50% methanol on ice. After centrifugation at 18,400 g for 15 min at 4°C, the supernatant was mixed with an equal volume of acetonitrile and subjected to centrifugation again. The supernatant was blown dry by nitrogen, dissolved in 100 μ l of 50% methanol, and used for further analysis. Concentrations of 22 kinds of bile acids, including primary and secondary BAs as well as their taurine conjugates, were determined by the UPLC-MS method (Yang et al., 2008; Zhang and Klaassen, 2010). Primary BAs analyzed included cholic acid (CA), chenodeoxycholic acid (CDCA), α-muricholic acid (αMCA), and β-muricholic acid (βMCA). Secondary BAs converted from corresponding primary BAs contained glycocholic acid (GCA) and deoxycholic acid (DCA) from CA; lithocholic acid (LCA) and ursodeoxycholic acid (UDCA) from CDCA; and murideoxycholic acid (MDCA), ω MCA, and hyodeoxycholic acid (HDCA) from αMCA and βMCA.

### Western blotting analysis

To examine the effect of FXR KO on the expression of proteins involved in neurotransmission, samples from hippocampus and cerebellum were homogenized, sonicated, and subjected to western blotting analysis. Total 30 mg proteins from each sample were separated on 12% SDS-PAGE. After transfer onto PVDF membranes, the proteins were incubated with respective primary antibodies against GABA transporter 1 (GAT1, cat# ab426), glutamate decarboxylase 2 (GAD65, cat# ab26113), GABA receptor subunits (GABA_A_Rα5, cat# ab9678; GABA_A_Rβ2/3, cat# MAB341), 5-HT receptor 1A (5-HT1A, cat# GTX104703), and norepinephrine transporter (SLC6A2, cat# sc-67216), and horseradish peroxidase conjugated secondary antibodies sequentially as described previously (He et al., 2013). The protein bands were visualized by an ECL-prime kit and quantified with Image J 1.46r software (NIH, USA).

### RT-PCR

Total RNAs from brain tissues of wild-type and FXR KO mice were extracted using Trizol according to the manufacturer's instructions (Life Technologies, MA, USA). After treatment with DNase I to eliminate trace amount of DNA contamination, the RNA samples were reversely transcribed into cDNA with Revert Aid First Strand cDNA Synthesis kit (Fermentas, MA, USA). Afterwards, the synthesized cDNA was used as templates for PCR reaction with respective primers: for FXR, 5′-TCTCTTTAAGTGATGACGGG-3′ and 5′-TTCTGCAGGGATGGAAACAT-3′; for GAPDH, 5′-ATGTGTCCGTCGTGGATCTGA-3′ and 5′-ATGCCTGCTTCACCACCTTCT-3′. PCR condition was set as following: 95°C for 5 min; then 35 cycles of 95°C for 30 s, 60°C for 30 s and 72°C for 30 s; finally 72°C for 5 min. PCR products were visualized by GeneGreen after electrophoresis on a 2% agarose gel.

### Statistical analysis

All data were presented as mean ± S.E.M. The difference between the two groups was evaluated by unpaired *t*-test. A value of *p* < 0.05 was regarded as statistically significant.

## Results

### Effect of FXR deletion on depressive-like behavior

**Figure 2:**
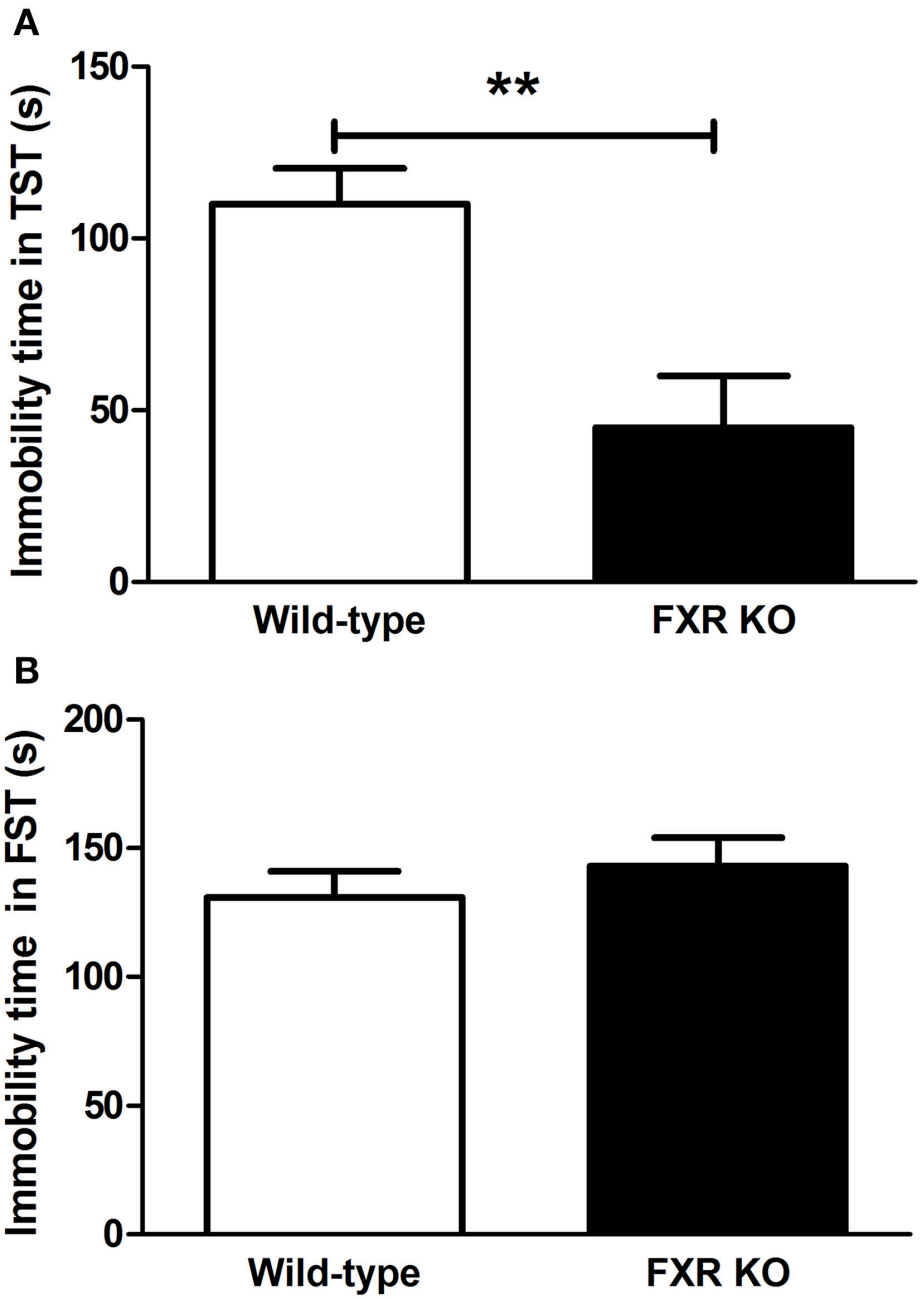
**FXR KO mice showed less depressive-like behavior**. In comparison with wild-type controls, FXR KO mice did not have reduced immobility time in the FST **(B)**, but exhibited significantly decreased immobility time in the TST **(A)**. *N* = 12/group; ** *p* < 0.01.

To determine the role of FXR on depression-like behavior, KO mice and their controls were subjected to the TST and FST. As shown in Figure 2A, in comparison with wild-type controls, FXR KO mice had significantly reduced immobility time [*t*_(20)_ = 3.426, *p* < 0.01] in the TST. Unexpectedly, in the FST the KO mice were not more active than the controls [Figure 2B; *t*_(22)_ = 0.799, *p* > 0.05]. Overall, results from these two tests reflect a possible anti-depressive effect of FXR deletion.

### Effect of FXR deletion on anxiety-related behavior

**Figure 3:**
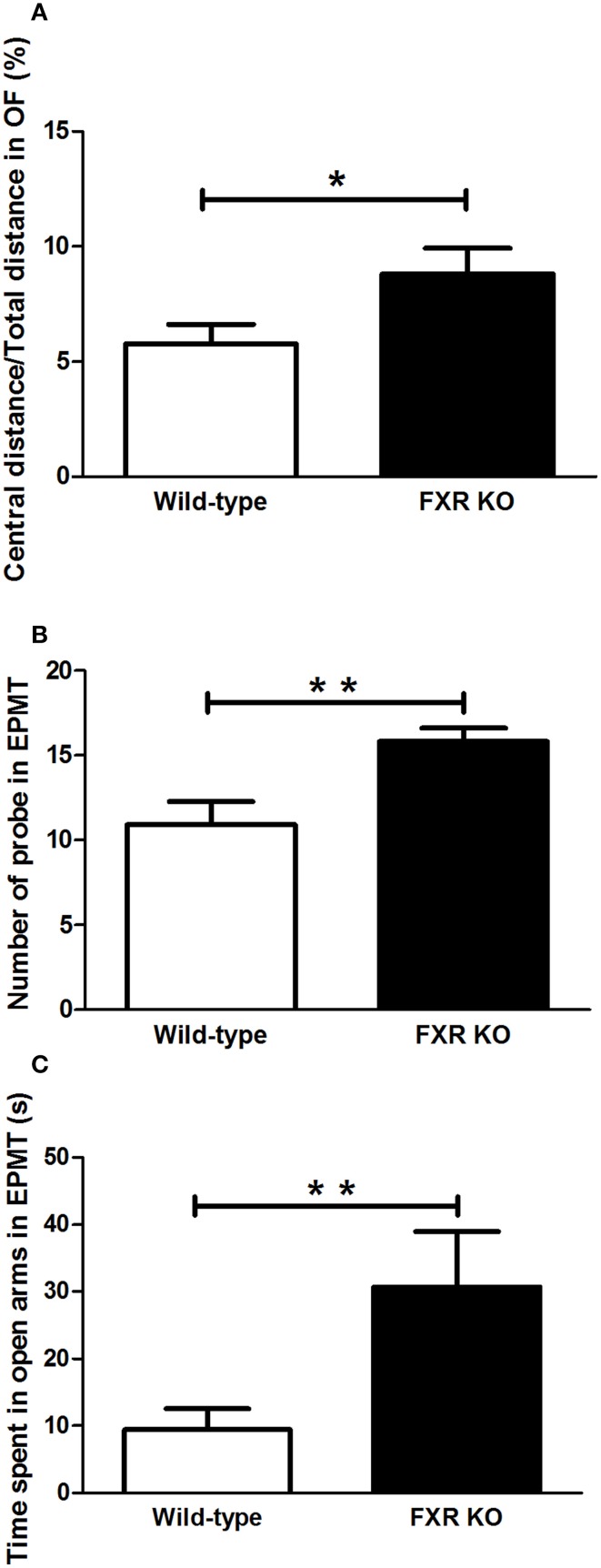
**FXR KO mice had low levels of anxiety-related behavior**. In comparison with wild-type controls, FXR KO mice had high ratio of central distance/total distance in the OFT **(A)**. Moreover, the KO mice had a greater tendency to explore **(B)** and to stay in the open arm in the EPMT **(C)**. *N* = 12/group; *p < 0.05; **p < 0.01.

In the two behavioral tests for anxiety, FXR deletion showed different behavioral effect on mice. In the OFT, FXR KO mice traveled more in the central area of the open field when compared with the controls [Figure 3A; *t*_(21)_ = 2.195, *p* < 0.05]. Similarly, in EPMT, mice with FXR deletion showed a greater tendency to enter the open arms to explore the new environment. The number of attempts to get into the open arms by FXR KO mice was significantly more than that of the controls [Figure 3B; *t*_(21)_ = 3.054, *p* < 0.01]. Moreover, once those KO mice entered the open arms of the elevated plus maze, they stayed there much longer than the controls [Figure 3C; *t*_(21)_ = 2.268, *p* < 0.05].

### Effect of FXR deletion on learning and memory

**Figure 4:**
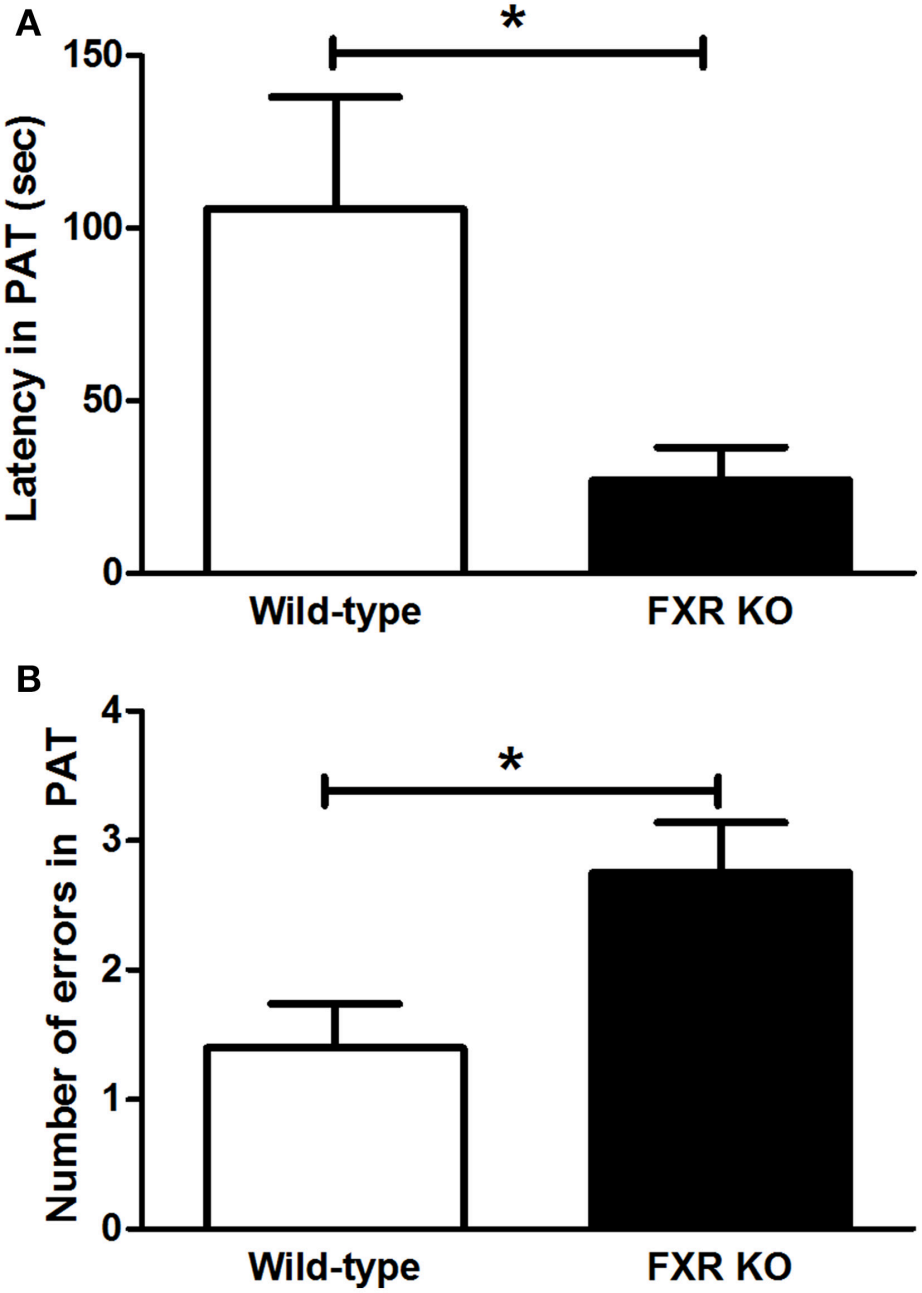
**FXR KO impaired cognitive ability of the mice**. In comparison with wild-type controls, FXR KO mice displayed decreased latency **(A)** but increased number of errors **(B)** in PAT. *N* = 12/group; * *p* < 0.05.

FXR deletion impaired the emotional memory of mice. In PAT, compared with the controls, the latency for FXR KO mice to enter the dark chamber on the test day was significantly shorter [Figure 4A; *t*_(21)_ = 2.231, *p* < 0.05]. Meanwhile, the number of entries into the dark chamber increased markedly in the KO mice [Figure 4B, *t*_(20)_ = 2.546, *p* < 0.05]. All these results indicate that the FXR deletion impaired the cognitive ability of mice.

### Effect of FXR deletion on motor performance

**Figure 5:**
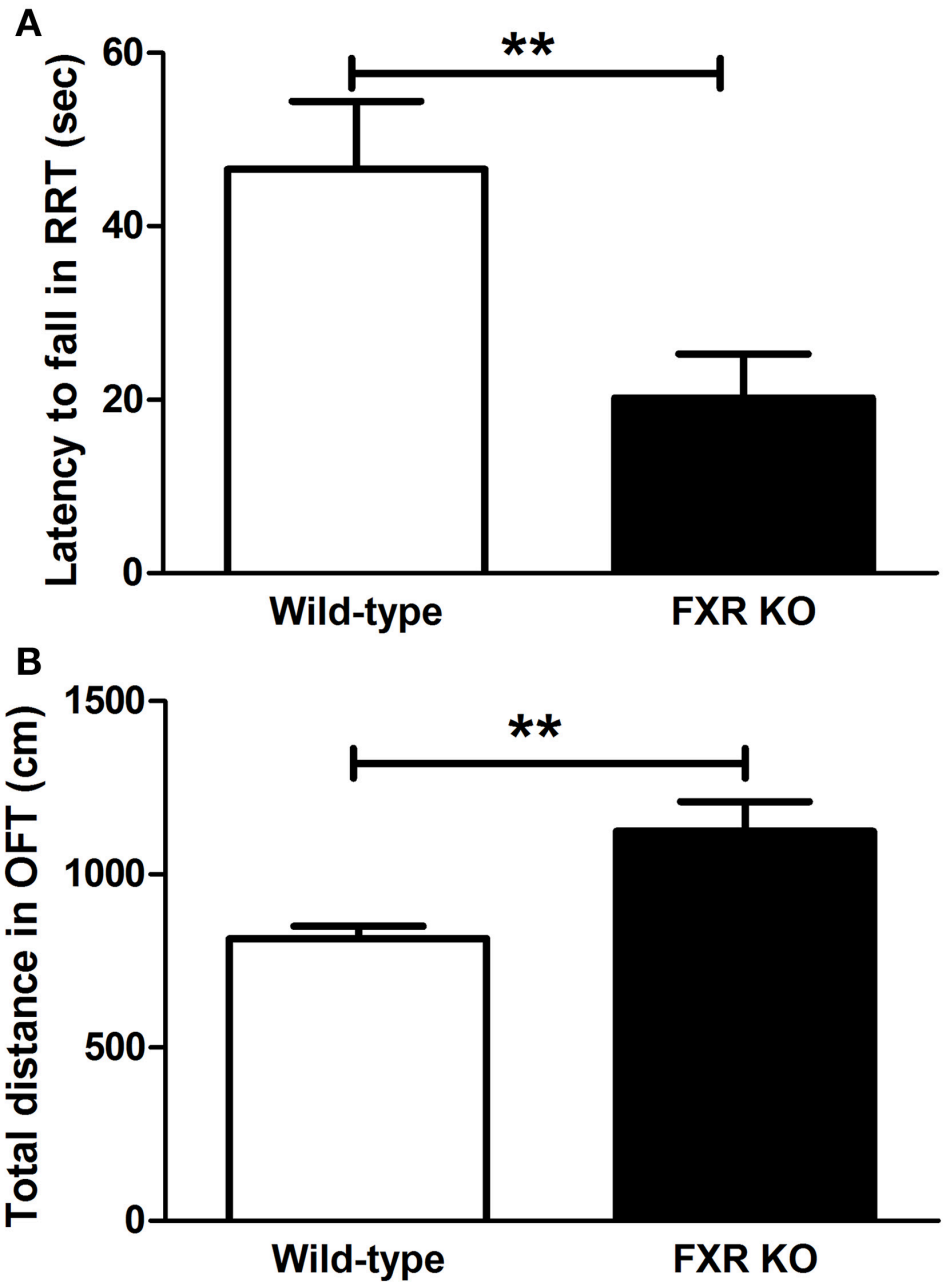
**FXR KO altered motor performance of the mice**. In comparison with wild-type controls, FXR KO mice fell from the rod more often in the RRT **(A)** but seemed to be hyperactive in the OFT **(B)**. *N* = 12/group; ** *p* < 0.01.

When FXR was deleted, the mice seemed to lose motor control. In the RRT, FXR KO mice lost their coordination of body and fell more easily from the rotating rods [Figure 5A; *t*_(20)_ = 2.927, *p* < 0.01]. However, KO mice appeared to be hyperactive. The total distance traveled in OFT was much longer in the KO mice than their wild-type controls [Figure 5B; *t*_(20)_ = 3.146, *p* < 0.01].

### Effect of FXR deletion on brain neurotransmitters

Since neurobiochemical signals in neurobehaviors such as mood, memory, and motor, are mediated by neurotransmitters in the CNS, seven common neurotransmitters in hippocampus, cerebellum, and prefrontal cortex were investigated by a LC-MS/MS method in this study. In the prefrontal cortex, none of the neurotransmitters examined showed any difference between FXR KO mice and their controls (Table 1). Similar results were seen in the hippocampus except for a significantly increased ratio of GABA to Glu [*t*_(22)_ = 2.887, *p* < 0.01]. However, in the cerebellum, the concentrations of GABA, NE, and HIAA were elevated prominently in FXR KO mice [*t*_(20)_ = 5.348, *p* < 0.001; *t*_(22)_ = 2.579, *p* < 0.05; and *t*_(22)_ = 3.594, *p* < 0.01, respectively] as well as the ratio of GABA to Glu [*t*_(20)_ = 3.469, *p* < 0.01]. In short, FXR deletion modulated neurotransmitter concentrations in different brain regions.

**Table 1:** **Comparisons of neurotransmitter concentrations in mouse hippocampus, cerebellum, and prefrontal cortex (mean ± S.E.M)**.

**Neurotransmitters**	**Hippocampus**		**Cerebellum**		**Prefrontal cortex**	
	**Wild-type**	**FXR KO**	**Wild-type**	**FXR KO**	**Wild-type**	**FXR KO**
GABA (μ g/g)	168.845 ± 21.772	171.059 ± 31.045	16.452 ± 0.680	20.717 ± 0.444^***^	53.232 ± 7.960	51.664 ± 4.475
Glu (μ g/g)	195.757 ± 27.440	156.079 ± 29.175	7.456 ± 0.530	7.821 ± 0.421	56.118 ± 9.310	50.784 ± 6.171
DA (ng/g)	47.757 ± 3.438	45.840 ± 4.431	N.D.	N.D.	N.D.	N.D.
NE (ng/g)	2216.645 ± 304.033	1791.320 ± 169.453	1542.997 ± 140.334	1911.069 ± 25.808^*^	8935.464 ± 876.937	7894.620 ± 912.644
Epi (ng/g)	33.290 ± 2.606	32.543 ± 2.992	N.D.	N.D.	N.D.	N.D.
5-HT (ng/g)	54.272 ± 7.440	55.936 ± 4.716	18.580 ± 3.684	21.735 ± 0.960	319.803 ± 34.737	310.598 ± 41.824
5-HIAA (ng/g)	532.423 ± 45.891	561.231 ± 51.504	165.938 ± 20.185	240.796 ± 7.137^**^	1158.452 ± 145.803	1269.219 ± 126.655
GABA/Glu	0.897 ± 0.057	1.149 ± 0.066^**^	2.135 ± 0.119	2.761 ± 0.132^**^	0.993 ± 0.056	1.062 ± 0.065
5-HIAA/5-HT	10.638 ± 0.763	10.310 ± 0.654	9.316 ± 0.800	11.329 ± 0.760	3.822 ± 0.460	4.477 ± 0.499

N = 12/group;

* p < 0.05;

** p < 0.01;

*** p < 0.001.

N.D., not detected.

### Effect of FXR deletion on proteins involved in neurotransmission

**Figure 6:**
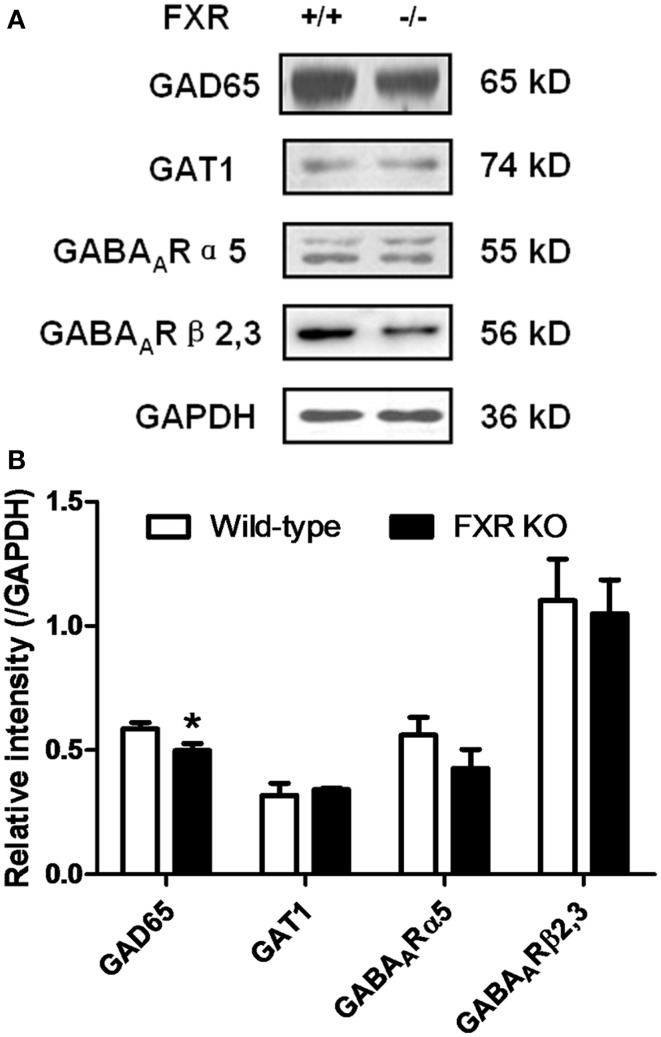
**FXR KO down-regulated hippocampal GAD65 of the mice. (A)** Western blotting analysis of GAD65, GAT1, GABA_A_Rα5, and GABA_A_Rβ2/3; **(B)** Gray intensity comparison. *N* = 8/group; * *p* < 0.05.

**Figure 7:**
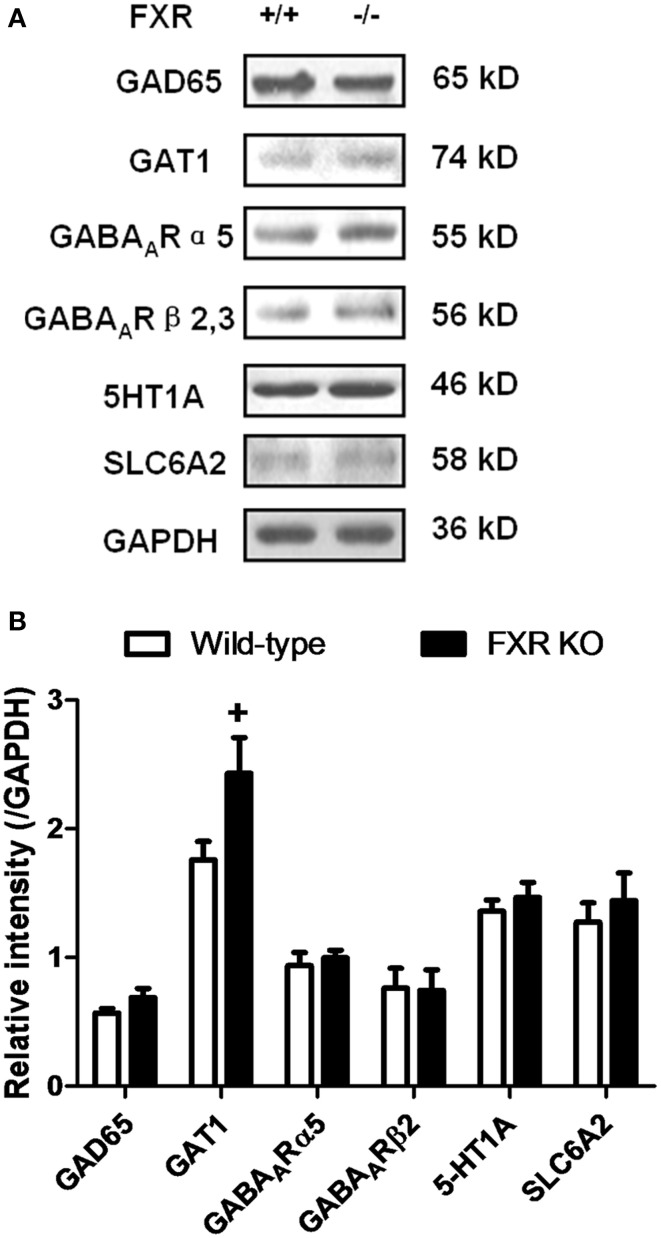
**FXR KO increased cerebellar GAT1. (A)** Western blotting analysis of GAD65, GAT1, GABA_A_Rα5, GABA_A_Rβ2/3, 5-HT1A, and SLC6A2. **(B)** Gray intensity comparison. *N* = 8/group; ^+^*p* < 0.1.

To examine whether FXR deletion affects the expression of proteins involved in neurotransmission, total proteins from the hippocampus and cerebellum of FXR KO mice and their controls were subjected to western blotting analysis. As shown in Figures 6A,B, FXR deletion did not change hippocampal expression of GAT1, and GABA_A_Rα5, and GABA_A_Rβ2/3. However, FXR KO mice showed down-regulated hippocampal GAD65 (*p* < 0.05). In the cerebellum, as shown in Figures 7A,B, most proteins examined were not altered, including GAD65, GABA_A_Rα5, GABA_A_Rβ2/3, 5-HT1A, and SLC6A2. The only exception was GAT1. FXR deletion tended to increase GAT1 in the cerebellum (*p* < 0.1).

### Effect of FXR deletion on serum and brain concentrations of bile acids

**Figure 8:**
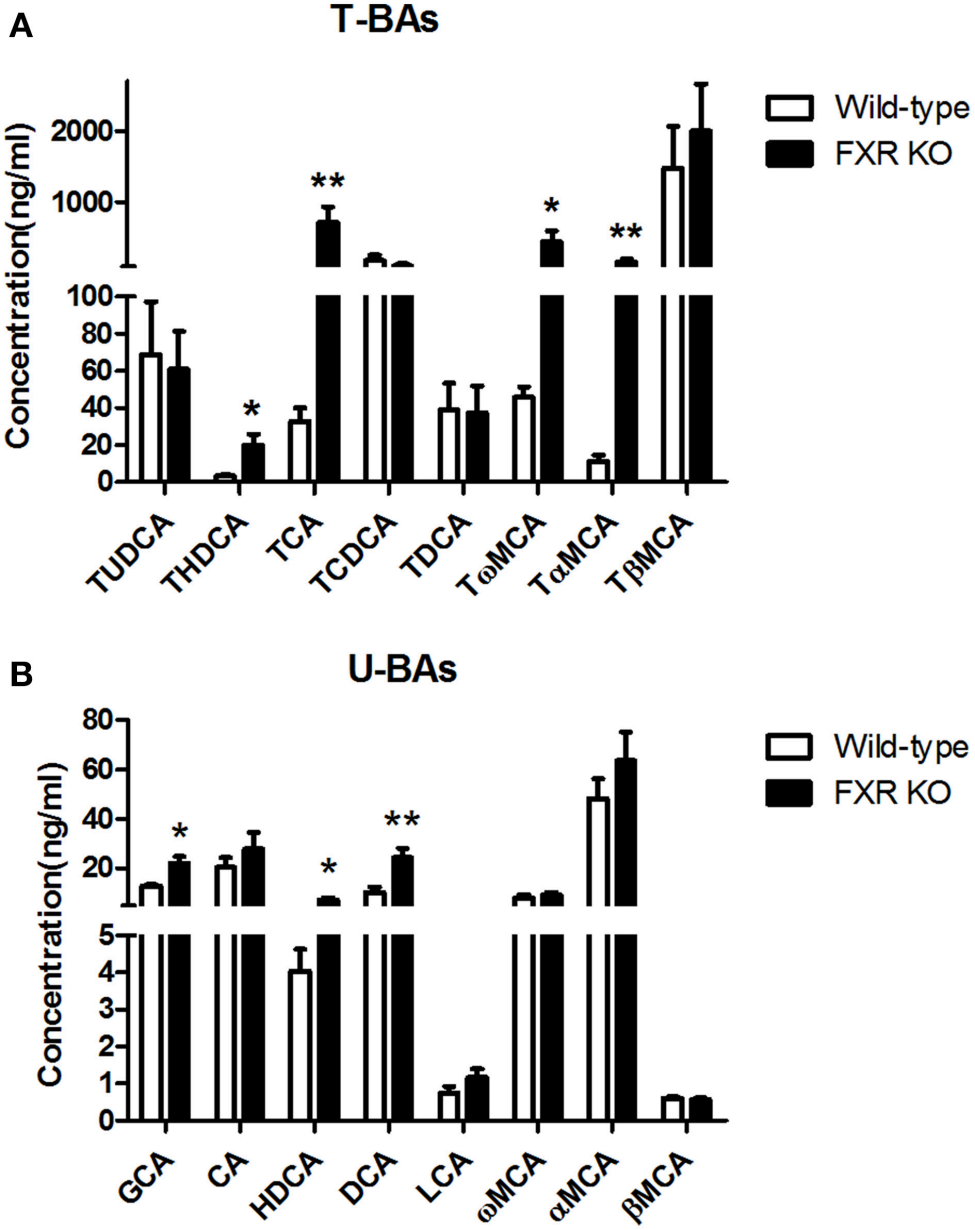
**FXR KO on BAs in serum**. In comparison with wild-type controls, FXR KO mice showed higher concentrations of THDCA, TCA, Tω MCA, and TαMCA in T-BAs in serum **(A)**; higher concentrations of GCA, HDCA, and DCA in U-BAs in serum **(B)**. *N* = 16/group; * *p* < 0.05; ** *p* < 0.01.

To determine the effect of FXR on the homeostasis of the peripheral bile acid system, serum concentrations of primary and secondary BAs were examined. However, six of them were too low to be detected. For the other 16 BAs, as shown in Figures 8A,B, FXR deletion led to up-regulation of THDCA [*t*_(26)_ = 2.314, *p* < 0.05], TCA [*t*_(24)_ = 2.463, *p* < 0.01], Tω MCA [*t*_(25)_ = 2.186, *p* < 0.05], TαMCA [*t*_(25)_ = 2.375, *p* < 0.01], GCA [*t*_(27)_ = 2.765, *p* < 0.05], HDCA [*t*_(28)_ = 2.319, *p* < 0.05], and DCA [*t*_(30)_ = 3.186, *p* < 0.01] without alteration of other kinds of BAs.

**Figure 9:**
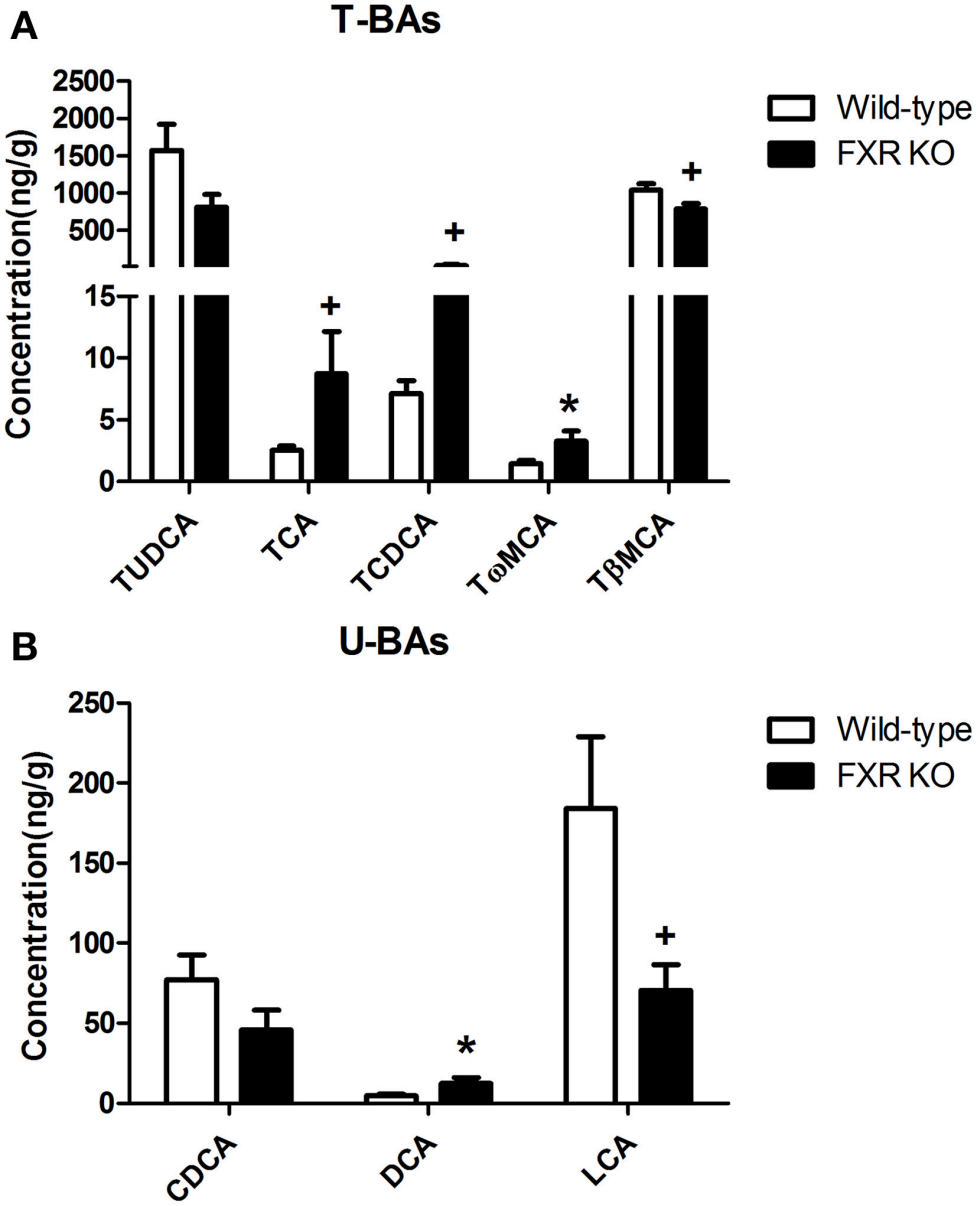
**FXR KO on BAs in brain**. In comparison with wild-type controls, FXR KO mice displayed higher concentrations of TCA, TCDCA, Tω MCA, and DCA **(A)** but lower concentrations of TβMCA and LCA in brain **(B)**. *N* = 7–10/group; * *p* < 0.05; ^+^*p* < 0.1.

By contrast, in brain, most of the BAs were not detectable. However, FXR deletion changed the relative concentrations of several brain BAs. As shown in Figures 9A,B, FXR deletion increased Tω MCA [*t*_(16)_ = 2.343, *p* < 0.05] and DCA [*t*_(14)_ = 2.339, *p* < 0.05]. Meanwhile, concentrations of TCA [*t*_(15)_ = 1.924, *p* = 0.0735] and TCDCA [*t*_(15)_ = 1.918, *p* = 0.0743] showed a tendency to increase. However, brain concentrations of TβMCA [*t*_(18)_ = 2.065, *p* = 0.0536] and LCA [*t*_(17)_ = 2.082, *p* = 0.0528] tended to decrease.

## Discussion

FXR modulates the synthesis and homeostasis of BAs. However, little is known whether FXR regulates neural circuitry or animal behavior. In the current study, we examined neural functions by comparing the behavior of FXR KO mice and their controls. Although *FXR* mRNA was undetectable in mouse brain, embryonic deletion of this otherwise ubiquitously expressed receptor altered many aspects of mouse behavior. FXR KO mice showed less depressive-like behavior in the TST and less anxiety-related behavior in the OFT and EPMT. These KO mice also had impaired memory in PAT and loss of locomotor coordination in the RRT but increased locomotor activity in the OFT. Further study showed that FXR deletion selectively altered the neurotransmitter profile in different brain regions. Moreover, FXR regulated protein expression associated with neurotransmission. All these results indicate that FXR plays an essential role in the maintenance of the homeostasis of neural transmission, although perhaps indirectly via modulation of homeostasis of BAs, thus influencing neurobehavior.

As expected, FXR KO showed less depressive-like and low levels of anxiety-related effects in mice in the respective behavioral tests. However, FXR deletion impaired cognitive function and motor coordination but enhanced locomotor activity. As mentioned previously, FXR itself is not present in mouse brain. Therefore, the CNS effects might be indirect. It is well-known that gene-knockout in mice may cause a compensatory change in comprehensive gene expressions and physiological activities, which in turn results in an indirect phenotypic effect on behavioral tasks (Stiedl and Meyer, 2003). As reduced body weight gain is seen in FXR KO mice aged from 15 to 39 weeks-old (Bjursell et al., 2013), the hyperactive locomotor activity observed in the current study might be in line with the increased energy expenditure of the animals. Moreover, although in both OFT and EPMT FXR KO mice showed less anxiety-related behaviors, it is not the only possible interpretation since several factors other than emotional behaviors such as locomotor activity, exploratory behavior, and behavioral motivation for novelty may confound with the anxiety-related behavior of mice (Bailey and Crawley, 2009).

To better understand the neural transmission underlying the abnormal behavioral response resulting from FXR deletion, the changes of seven common neurotransmitters in several brain regions were examined. Disruption of the neurotransmitter homeostasis in certain brain regions is known to cause serious neurological dysfunction. For instance, excessively increased excitatory Glu but reduced inhibitory GABA is closely associated with pathophysiology of depression (Sanacora et al., 1999). Drugs facilitating glutamatergic transmission may enhance memory (Staubli et al., 1994). Similar effect can be obtained by antagonizing GABA transmission (Kim et al., 2014). Nevertheless, a disrupted balance between excitatory and inhibitory neurotransmitter systems is central to the pathogenesis of a variety of neurological and psychiatric disorders (Chen et al., 2010; Cherlyn et al., 2010; Niswender and Conn, 2010; Yuksel and Ongur, 2010; Luscher et al., 2011).

In our experiments, several neurotransmitters in both hippocampus and prefrontal cortex, regions implicated in memory and emotion, were examined. To our surprise, none of the neurotransmitters analyzed showed significant alterations in these two regions. However, the balance of GABA to Glu was disturbed since it was remarkably elevated in the hippocampus of the FXR deleted mice. This might partly explain the enhanced anti-depressive and anxiolytic but impaired cognitive behaviors of FXR deleted mice. The neurotransmitters in the cerebellum of both FXR KO and their controls were also examined, since cerebellum is the major brain area controlling locomotor activity as well as cognitive functions such as attention (Wolf et al., 2009). In the cerebellum of FXR KO mice, not only was the balance of GABA/Glu disturbed, but also the concentrations of NE and 5-HIAA were greatly increased. Decrease of cerebellar NE has been reported to be accompanied by a concomitant increase of motor deficit (Bueno-Nava et al., 2008; Yokota et al., 2013). 5-HIAA is a major metabolite of 5-HT and its elevation reflects enhanced serotonergic activity. Abnormally increased cerebellar 5-HT has been indicated in rats with motor deficit (Bueno-Nava et al., 2010). Therefore, the abnormal motor performance of FXR KO mice might be due to disturbed norepinephrinergic and serotonergic transmissions as well as the GABAergic and glutamaergic ones in cerebellum.

As the equilibrium of GABA, NA, and 5-HT were disturbed in the hippocampus and cerebellum of FXR KO mice, we further investigated whether these alterations changed the expression of proteins involved in the synthesis, transport, and signaling of these neurotransmitters. As shown in Figures 6,7, most proteins examined, including GABA_A_Rα5, GABA_A_Rβ2/3, 5-HT1A, and SLC6A2, were unchanged by FXR deletion. However, hippocampal GAD65 was decreased while cerebral GAT1 was increased. GAD65 is highly enriched in axon terminals and is associated with synaptic vesicles. Its expression is modulated at the transcriptional level by a short-term mechanism. GAD65 knockout mice display electrophysiological features consistent with a reduced vesicular release of GABA (Asada et al., 1996). GAT1, one of the GABA transporters exclusively expressed in the brain, participates in regulating the synaptic overspill of GABA and activation of GABA receptors at extrasynaptic loci (Dalby, 2003). Therefore, FXR KO mice showed interference in GABA transport and signaling in both cerebellum and hippocampus, leading to alterations of neurobehavior.

Interestingly, the FXR KO mice showed decreased attention, hyperactivity, and hyper-reactivity to stress, which partially resembles the attention deficit hyperactivity disorder (ADHD) in human. Furthermore, the increased NE in the cerebellum of FXR KO mice was also found in an animal model of ADHD (de Villiers et al., 1995). They therefore have good face validity (hyperactivity and learning difficulties), but lack constructive and predictive validity (van der Kooij and Glennon, 2007). All these findings raise the possibility that FXR might be involved in the pathophysiology of a subset of ADHD.

As discussed above, FXR actively participates in the modulation of cholesterol metabolism (Sinal et al., 2000). Although cholesterol itself does not cross the BBB, its oxygenated product, 27-hydroxycholesterol, can be transported through the BBB (Bjorkhem, 2006), thereby influencing neurotransmission and neurobehavior. Moreover, circulating serum bile acids such as DCA and CDCA, the derivatives of hepatic cholesterol, can increase the permeability of BBB during obstructive cholestasis via disruption of tight junctions (Quinn et al., 2014). In addition to signaling through FXR, bile acids such as LCA can activate the preganane X receptor (PXR) and vitamin D receptor (VDR) (Hylemon et al., 2009), both of which are expressed within the CNS (Eyles et al., 2014; Jain et al., 2014). In our experiments, FXR deletion caused significant elevation of circulating THDCA, TCA, DCA, TαMCA, Tω MCA, GCA, and HDCA accompanied by alterations of several BAs in brain. It is reasonable to postulate that the peripheral elevation of these BAs altered the homeostasis of neurotransmission via regulation of BBB permeability and activation PXR, VDR or certain unknown receptors in the CNS. However, at the present state of knowledge, further investigation is needed to corroborate this hypothesis.

Overall, FXR deletion altered BA concentrations in blood and changed the compositions of BA in brain, partially related to the interactions of BA's with the BBB. This resulted in alterations of neurotransmitters and changed motor activity, cognitive function, and mood of the FXR KO mice. The neurobehavioral changes of the FXR KO mice represent an unique case of mild hepatic encephalopathy and the differential susceptibility of different CNS functional domains.

### Conflict of interest statement

The authors declare that the research was conducted in the absence of any commercial or financial relationships that could be construed as a potential conflict of interest.

## Supplementary material

Supplementary Figure 1 **FXR mRNA expression pattern in brain of wild-type mouse**. Liver served as positive control.
