# Supplementary figures and images for: Deletion of mouse FXR gene disturbs multiple neurotransmitter systems and alters neurobehavior
Source: Front Behav Neurosci. 2015 Mar 30;9:70. doi: 10.3389/fnbeh.2015.00070 (PMC4378301; doi:10.3389/fnbeh.2015.00070)

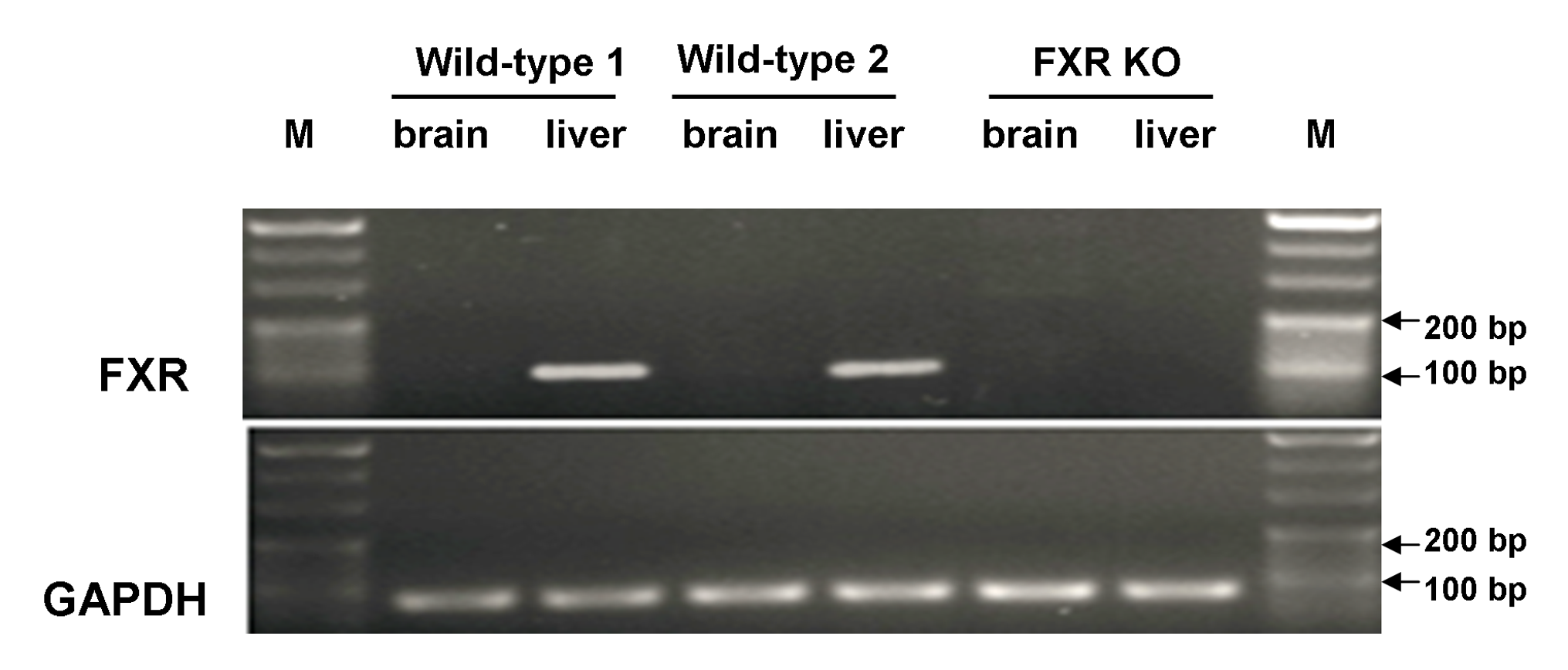

Supplement: Supplementary Figure 1 — FXR mRNA expression pattern in brain of wild-type mouse. Liver served as positive control. [file Image1.TIF]
